# Comparison of tissues (heart vs. brain) and serological tests (MAT, ELISA and IFAT) for detection of *Toxoplasma gondii* in naturally infected wolverines (*Gulo gulo*) from the Yukon, Canada

**DOI:** 10.1016/j.fawpar.2019.e00046

**Published:** 2019-03-08

**Authors:** Rajnish Sharma, Sarah Parker, Batol Al-Adhami, Nicholas Bachand, Emily Jenkins

**Affiliations:** a Department of Veterinary Microbiology, Western College of Veterinary Medicine, University of Saskatchewan, 52 Campus Drive, Saskatoon, Saskatchewan S7N 5B4, Canada; b Centre for Applied Epidemiology, Large Animal Clinical Sciences, Western College of Veterinary Medicine, University of Saskatchewan, 52 Campus Drive, Saskatoon, Saskatchewan S7N 5B4, Canada; c Centre for Food-borne and Animal Parasitology, Canadian Food Inspection Agency, Saskatoon Laboratory, 116 Veterinary Road, Saskatoon, Saskatchewan S7N 2R3, Canada

**Keywords:** *Toxoplasma gondii*, MAT, ELISA, IFAT, MC-qPCR

## Abstract

Toxoplasmosis is an important parasitic zoonosis worldwide. Many human and animal surveys use serological assays based on *Toxoplasma gondii* antibody detection in serum, a matrix that is not routinely available from wildlife. Commonly used serological assays have rarely been validated for use with fluids other than serum, nor validated for their performance in wildlife species. New molecular assays, such as magnetic capture DNA extraction and real-time PCR (MC-qPCR), offer high sensitivity for detection of *T. gondii* DNA in tissues. The aims of this study were to (1) assess prevalence of *T. gondii* DNA based on MC-qPCR detection in brain and heart of naturally infected wolverines (*Gulo gulo*) from the Yukon, Canada (2) compare two matrices [heart fluid (collected from thawed heart) and filter eluate (eluted from blood soaked filter paper)] for antibody detection in the same species, and (3) evaluate the performance of three serological tests [modified agglutination test (MAT), enzyme linked immunosorbent assay (ELISA) and indirect fluorescent antibody test (IFAT)] to detect naturally infected wolverines as determined by MC-qPCR. DNA of *T. gondii* was detected in heart and/or brain in 16 of 68 wolverines (24%, 95% CI: 15.0–34.8). Tissue prevalence and infection intensity was higher in heart [16 positives, mediantachyzoites equivalents per gram (TEG) =1221] compared to brain (10 positives, median TEG = 347). Heart fluid (HF) and filter eluates (FE) performed equally well in ELISA and IFAT in terms of relative sensitivity, but HF performed better with MAT. ELISA and IFAT had higher relative sensitivity (94%) and relative specificity (100%) compared to MAT (relative sensitivity 75% and relative specificity 92%). Overall, our findings indicate that the parasite burden in naturally infected wolverines was higher in heart compared to brain, heart fluid performed better than filter paper eluate for serological testing using MAT, and both IFAT and ELISA had higher relative sensitivity, relative specificity, and accuracy compared to MAT.

## Highlights

• The infection intensity of *T. gondii* in naturally infected wolverines was higher in heart compared to brain.
• Heart fluid performed better than filter paper eluate for serological testing using MAT.
• Both IFAT and ELISA had higher sensitivity, specificity, and accuracy compared to MAT.
• We suggest evaluating the performance of commercially available kits before their usage to detect *T. gondii* antibodies.

## Graphical abstract

Serological tests for detection of *Toxoplasma gondii* exposure in naturally infected wolverines Wolverine picture supplied by Emilie Bouchard (University of Saskatchewan).

**Figure:**
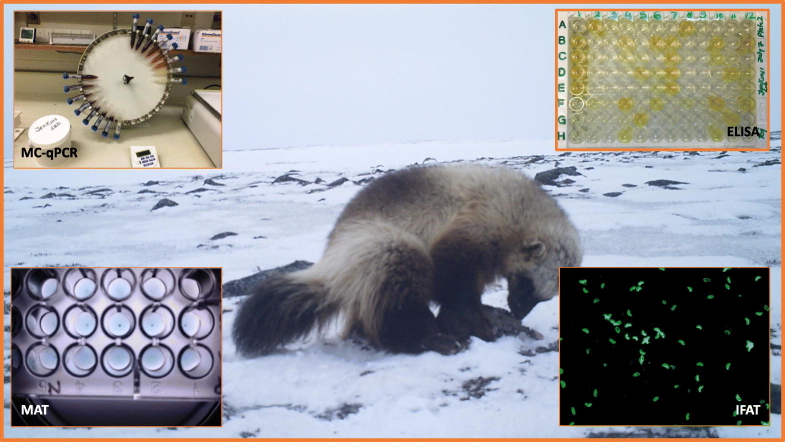

## 1 Introduction

*Toxoplasma gondii* infects almost all warm-blooded animals including humans, mammals and birds; one third of the global human population shows evidence of exposure to *T. gondii* (Dubey, 2010; Hill and Dubey, 2002; Montoya and Liesenfeld, 2004). Routes of transmission to people are (1) consumption of food or water contaminated with oocysts shed by felids, the only final hosts, (2) consuming raw/undercooked meat of infected animals containing bradyzoites in tissue cysts, and (3) via transmission of tachyzoites vertically from mother to fetus and (4) horizontally during blood transfusion and organ transplant (Dubey, 2010; Hide, 2016; Hill and Dubey, 2002; Robert-Gangneux and Darde, 2012; Ryning et al., 1979; Tenter et al., 2000). Therefore, animals play a key role not only in the life cycle of the parasite but also as a source of infection for people.

In northern Canada, seroprevalence of *T. gondii* varying from 6 to 42% has been reported in wild animals (Bachand et al., 2018; Elmore et al., 2016; Reichard et al., 2008; Simon et al., 2011); however, to the best of our knowledge, no reports are available on the prevalence in the Yukon. Wild carnivores can act as sentinel animal species of *T. gondii* (Bachand et al., 2018; Burrells et al., 2013) and can indicate whether *T. gondii* is present in the surrounding environment. Wolverines (*Gulo gulo*), fur-bearing mesocarnivores, have the potential to act as a sentinel animal host species, and a previous study showed that 42% of the wolverines (n = 41) were seropositive to *T. gondii* in Nunavut, Canada based on the modified agglutination test (MAT) (Reichard et al., 2008).

Methods that directly detect the parasite or its DNA in tissue indicate active infection in animals. These methods include bioassays, tachyzoite culture, immunohistochemistry and molecular techniques like PCR (Liu et al., 2015). Cat and/mouse bioassays are the gold standard tests. Cat bioassay is a highly sensitive test as it can use a larger volume of tissue (500 g or more); however, it is time consuming, expensive and has ethical issues (Jurankova et al., 2014; Liu et al., 2015; Robert-Gangneux and Darde, 2012). Moreover, bioassays are not useful for previously frozen samples since testing relies on parasite viability (El-Nawawi et al., 2008), are not feasible for large-scale screening (Jurankova et al., 2015) and do not quantify infection intensity. A recently developed magnetic capture- qPCR (MC-qPCR), capable of analyzing up to 100 g of tissue (Opsteegh et al., 2010) has successfully been used to detect *T. gondii* DNA in wild animals (Bachand et al., 2018). Thus, we selected MC-qPCR over conventional PCR methods due to higher sensitivity (it analyses 100 g vs 25–200 mg of tissue) and specificity (*T. gondii* DNA is selectively extracted using a specific probe bound to magnetic beads) (Opsteegh et al., 2010). Heart and brain are the most common predilection sites for *T. gondii* in animals (Gisbert Algaba et al., 2017; Gisbert Algaba et al., 2018; Jurankova et al., 2014; Koethe et al., 2015), but this has not yet been established for wolverines. Therefore, the first objective of the current study was to determine whether brain or heart is a more preferred site for *T. gondii* using MC-qPCR.

Indirect methods of detection are serological tests, which could indicate only exposure to *T. gondii* (Gisbert Algaba et al., 2017). For serological tests, serum is the specimen of choice, but it is not always feasible to obtain serum samples from live or dead wild animals (Aston et al., 2014; Curry et al., 2011; Curry et al., 2014). Several substitutes for serum in wild animal surveys primarily include blood, blood or body cavity fluid on filter paper, and meat juice (Aston et al., 2014; Bachand et al., 2018; Curry et al., 2011; Elmore et al., 2014); however, each matrix has its own merits and demerits. For example, collecting blood or cavity fluid on filter paper is preferred in remote areas where storage facilities are not available (Curry et al., 2011; Curry et al., 2014). Examining blood/cavity fluid or meat juice rather than filter paper eluate may be advantageous in terms of detecting low antibody levels. Meat juice and filter eluate have been used in several wildlife studies for detection of anti-*T. gondii* antibodies (Aston et al., 2014; Bachand et al., 2018; Curry et al., 2011; Elmore et al., 2014). Comparative studies on the performance of heart fluid (a mix of meat juice and serum) vs. filter eluate in wild animals are not available. Therefore, our second objective was to compare heart fluid and filter eluate collected from wolverines using three serological tests, namely MAT, enzyme-linked immunosorbent assay (ELISA), and indirect fluorescent antibody test (IFAT).

Commercially available kits for the detection of anti-*T. gondii* antibodies are generally not validated for use in wildlife. Therefore, their performance in the target species should be evaluated before their use in sero-surveys. Previously, in-house MAT had been used in a *T. gondii* survey in wolverines in Nunavut; 1:25 cut off was used without any validation (Reichard et al., 2008). Therefore, the third objective of the current study was to compare the performance of MAT, ELISA and IFAT to detect anti-*Toxoplasma gondii* antibodies in naturally exposed/infected wolverines relative to MC-qPCR. Our findings will be helpful for wildlife disease researchers, and public health practitioners to select tissues, serological specimen and tests for future wildlife as well as public health surveys on *T. gondii*.

## 2 Methodology

### 2.1 Wildlife samples

Sixty-eight wolverine carcasses, submitted by Yukon fur trappers to Environment Yukon, Canada were necropsied. For each wolverine, whole heart and brain were placed individually in double plastic bags to avoid cross-contamination between samples, and bags were labelled with wolverine ID number. Thoracic fluids were collected on five Nobuto filter paper strips (Advantec MFS, Inc., Dublin, CA, USA). After allowing to dry at room temperature overnight, filter paper strips were kept in individual envelopes, and stored at -20 °C. The samples were shipped to the Department of Veterinary Microbiology at the University of Saskatchewan, Canada and stored at −20 °C until further analysis.

#### 2.1.1 Collection of heart fluid (HF)

One ml of accumulated fluid was collected from the bags containing thawed hearts, and centrifuged at 1000*g* for 5 min at 4 °C. The supernatant was collected in a 1.5 ml Eppendorf tube, and stored at -20 °C. As we collected whole heart, and did not open them before collecting heart fluid, the heart fluid may have some proportion of serum from the clotted blood present in the heart.

#### 2.1.2 Elution of Nobuto filter paper

The blood soaked areas of two Nobuto filter paper strips were cut into 7–8 pieces, placed in an Eppendorf tube containing 800 μl of Dulbecco's Phosphate Buffered Saline and kept at 4 °C for 16 h (Curry et al., 2011). The resulting filter eluate (FE) was collected in a new labelled Eppendorf tube and stored at -20 °C.

### 2.2 Detection of *T. gondii* DNA in tissues using MC-qPCR

#### 2.2.1 Extraction of *T. gondii* DNA

The whole heart and brain were thawed at 4 °C for 24 h. Then, connective tissue and fat were removed using a new scalpel blade and forceps for each organ. Magnetic capture-DNA extraction method was used to extract DNA from the whole heart and brain individually (Opsteegh et al., 2010). For each batch of DNA extraction, two spiked beef samples (2.5 × 10^5^ tachyzoites/100 g and 2.5 × 10^6^ tachyzoites/100 g) were used as positive controls and a beef sample without spiking was used as a negative control. Tachyzoites of *T. gondii* (VEG type III) were obtained from the Centre for Food-borne and Animal Parasitology (CFAP), Saskatoon. The DNA was stored at —20°C until further use.

#### 2.2.2 Detection and quantification of *T. gondii* DNA

Real-time PCR was conducted in a thermocycler (Biorad, Hercules, California, USA) following the exact protocol as established by (Bachand et al., 2018). In brief, each reaction of 25 μl contained 0 .5M iTaq DNA polymerase (Biorad, CA, USA), 10 μM of each primer (Tox 9F and Tox 11 R), 5 μM of a competitive internal amplification control (CIAC) probe, 2 fg of CIAC, 6.75 μl of PCR grade water and 8 μl template DNA. All reactions were run in duplicates; for each run, positive and negative extraction controls as well as no template controls were included. A dilution series of *T. gondii* plasmid DNA for obtaining a standard curve was also included in each run (Bachand et al., 2018). The reaction was considered as positive if (1) the C*p* value was less than or equal to 35, (2) the positive control was positive, and (3) the negative and no template controls were negative. Samples with Cp values between 35 and 40 were considered positive if a 188 bp band was identified on gel electrophoresis. In case one of the duplicates or the CIAC did not amplify, the sample was repeated.

From the Cp values, quantification of parasites was done using the formula (Bachand et al., 2019): log10 (tachyzoites) = (43.3–Cp) /3.07).

Quantification of parasites was expressed as number of tachyzoite-equivalents (TE) and termed as infection intensity.

### 2.3 Detection of *T. gondii* antibodies

#### 2.3.1 Modified agglutination test (MAT)

The modified agglutination test was performed using a commercially available kit (New Life Diagnostic LLC, Carlsbad, CA, United States). Results were interpreted as per manufacturer's instructions.

#### 2.3.2 Enzyme linked immuno sorbent assay (ELISA)

Using commercially available ID Screen® Toxoplasmosis Indirect Multi-species kits (IDvet, Grabels, France), ELISA was performed as per manufacturer's instructions. Optical density (O.D.) values at 450 nm were recorded using an ELISA automated plate reader (Spectramax, Molecular Devices). S/P % was calculated by formula S/P% =  (OD _sample_-OD _negative control_/OD _positive control_-OD _negative control_) × 100. Samples presenting an S/P % less than or equal to 40% were considered negative. Samples with an S/P ratio greater than or equal to 50% were considered positive. If the S/P ratio was between 40% and 50%, test outcome was considered doubtful and repeated.

#### 2.3.3 Indirect fluorescent antibody test (IFAT)

The IFAT was performed using commercially available antigen-coated Teflon-masked slides from VMRD (Pullman, WA, USA) and protein A/G FITC conjugate (BioVision, Milpitas, CA, USA). For IFAT, the optimal concentration of protein A/G FITC-conjugate was determined by using its different dilutions: 1: 300, 1:600 and 1:1200 with different dilutions of heart fluid and filter eluate. The staining procedure was performed as per manufacturer's instructions and the slides were observed using a fluorescent microscope at 40-100×. The wells with diffuse or intact peripheral staining of tachyzoites were considered positive. The wells with only apical/no staining of tachyzoites were considered negative. The broken peripheral staining of tachyzoites was considered as doubtful and was repeated.

#### 2.3.4 Assessment of test specificity

Each MAT and ELISA included positive and negative controls provided by the manufacturers. Serum samples positive and negative for anti-*T. gondii* antibodies obtained from experimentally infected pigs were used in each IFAT test. To check for cross-reactivity, serum samples that were positive and negative for antibodies to *Neospora caninum* (bovine) and *Hammondia hammondi* (feline) were examined in each serological test. Reference positive and negative sera (for *T. gondii, H. hammondi* and *N. caninum*) were provided by the CFAP, Saskatoon.

#### 2.3.5 Pilot study: evaluation of serological test performance with wolverine samples

As commercially available kits and slides used in this study were not evaluated for their use in wolverines and neither for use with filter eluates, we conducted a pilot study with three objectives 1) to evaluate if cut-off values (MAT) and sample dilutions (ELISA) as per manufacturer's recommendation work for this animal host species, 2) to establish cut-off values for IFAT as the manufacturer provided no cut-off values, and 3) to compare HF and FE to detect antibodies to *T. gondii*. We selected nine animals for this pilot study: three wolverines were negative in MC-qPCR for both tissues (group-1 N, negative). Six wolverines were MC-qPCR positive for both tissues, and all had Cp value <30 for heart tissue. Three of the latter 6 had a Cp value >30 for brain (group-2 LP, low positive), and the other three had a Cp value <30 for brain (group-3, HP, highly positive). The MAT was performed using HF and FE with four dilutions [1:25 (recommended cut-off), 1:50, 1:100 and 1:200]. ELISA was performed using HF and FE with two dilutions [1:2 (recommended dilution for HF) and 1:4] as well as with no dilution. IFAT was performed using HF and FE with five dilutions (1:2 to 1:32) and the results were graded 1+ to 3+ based on fluorescence intensity.

### 2.4 Statistical analysis

#### 2.4.1 Comparison of heart and brain

The proportion of positive results for heart and brain samples were compared with techniques for matched samples. McNemar Chi-square tests were performed to compare the proportion of wolverines positive for *T. gondii* DNA from hearts and brains. The kappa value (k) was calculated to determine the agreement between detection using the heart and brain. As the dependent variable (infection intensity) was not normally distributed, we used the Wilcoxon signed rank test (suitable for paired data) to compare levels of TE in heart and brain.

#### 2.4.2 Comparison of serological methods

All doubtful results on serological tests were considered negative in this study. Using MC- qPCR as the determinant of infection status, relative sensitivity, relative specificity, positive predictive value, negative predictive value and accuracy were calculated for each serological test. The kappa value (k) was calculated to determine the agreement between detection of anti-*T. gondii* antibodies using MAT, ELISA and IFAT. Kappa values of ≤0.40, 0.40–0.60, 0.61–0.80 and ≥ 0.81 were considered to represent slight to poor, moderate to good, substantial and excellent agreement, respectively (Viera and Garrett, 2005).

All statistical tests were performed using commercial statistical software (IBM SPSS, ver. 24; Armonk, New York, USA).

### 2.5 Ethical approval

As the animals were harvested for purposes other than research, this was considered Category A and exempt from Animal Research ethics review at the University of Saskatchewan. We worked closely with Government of the Yukon partners for wildlife research and export permits.

## 3 Results

### 3.1 Comparison of heart and brain

Out of the 68 wolverines tested, 16 (24%) were positive for *T. gondii* DNA in at least one tissue. Fifty-two wolverines were negative for *T. gondii* in both types of tissue. Ten wolverines were positive for *T. gondii* DNA in both heart and brain, and six were positive in heart tissue only. The proportion of positive hearts (23.5%, 16/68) and brains (14.7%, 10/68) was significantly different (P = 0.031, McNemar test).

There was substantial agreement between the detection of *T. gondii* from the hearts and brains (k = 0.72). The infection intensity in the hearts (n = 16) varied from 0.35 to 70,624 tachyzoites equivalents per gram (TEG) (median = 1221, 25th –75th percentiles = 186 to 7157), and in the brains (n = 10) from 0.91 to 10,755 TEG (median = 347, 25th –75th percentiles = 37.6–721.0). Of the 10 animals positive in both tissues, eight wolverines had higher TEG in heart than brain, whereas two had higher TEG in their brain. The hearts had a significantly (P = 0.047, Wilcoxon Signed Rank test, n = 16) higher median parasite burden than the brains.

### 3.2 Specificity of serological assays

We observed cross-reactivity with serum samples positive for *H. hammondi* antibodies using MAT; however, no cross-reactivity was observed with serum samples positive for *N. caninum* antibodies using MAT, and serum samples positive for *H. hammondi* or *N. caninum* antibodies using ELISA or IFAT.

### 3.3 Results of the pilot study

All 3 MC-qPCR negative wolverines (group-1 N) were negative at all dilutions in all three tests, except for ELISA, where undiluted heart fluid and filter eluate from one of the wolverines (ID-18) reacted positively. We observed no discordance in qualitative (sero-positivity) and quantitative (titer) results in group-3 (HP) using the MAT, but in group- 2 (LP) the MAT titer for HF was higher than for FE, and one MC-qPCR positive wolverine (ID-33) showed a negative result with FE, indicating that HF may be a better specimen for the MAT and that a cut off value of 1:25, as recommended by the manufacturer, can be used for HF.

Using ELISA in groups 2 (LP) and 3 (HP), there was no difference when HF and FE were compared at dilutions of 1:2 and 1:4. However, S/P% was consistently higher for HF than for FE (at both dilutions). The observed S/P% of HF was also higher at a dilution of 1:2 than at 1:4. Therefore, HF and FE can be used at the recommended dilution of 1:2 for ELISA, and HF is a better matrix than FE for detecting anti-*T. gondii* antibodies. Using IFAT in groups 2 (LP) and 3 (HP), results were similar between HF and FE at dilutions of 1:2 and 1:4, but fluorescence was more intense for HF than FE (at both dilutions) for two positive wolverines (IDs-52 and 56). At dilutions of 1:8 and 1:16, HF and FE were still positive, but fluorescence decreased to 1+. At a dilution of 1:32, the FE of two wolverines (IDs-52 and 56) was negative (data not shown in table). The results of IFAT indicated that the cut-off values of 1:2 and 1:4 can be used for both HF and FE. Results are summarized in Table 1.

**Table 1:** Comparison of heart fluid and filter eluate using serological methods (MAT, ELISA and IFAT) relative to magnetic capture qPCR.

Group	ID	MAT titer Cut-off 1:25		ELISA S/P% Cut-off 50% S/P					IFAT Cut-off 1+			
		HF	FE	HF	FE	HF	FE	HF	HF	FE	HF	FE
				UD^$^	UD	1:2	1:2	1:4	1:2	1:2	1:4	1:4
1 N	18	N	N	64.2	54.6	17.8	12.8	15.3	N	N	N	N
	38	N	N	27.5	33.3	8	4.7	7.8	N	N	N	N
	72	N	N	9.5	13.6	5.8	1.7	7.9	N	N	N	N
2 LP	12	1:100	1:50	262.8	238	236.8	229.3	225.9	3+	3+	3+	3+
	33	1:25	N	209.7	177.5	172.7	148.2	149.7	3+	3+	3+	3+
	56	1:200	1:100	277.3	238.8	262.8	228.4	244.7	3+	2+	3+	2+
3 HP	9	1:200	1:200	272	248.4	262.3	241.8	240.9	3+	3+	3+	3+
	34	1:50	1:50	223.9	242.9	204.7	200.2	188.4	3+	3+	3+	3+
	52	1:200	1:200	254	256.1	245.1	241.8	229.2	3+	2+	3+	2+

1 N = negative on MC-qPCR, 2 LP = positive on MC-qPCR on heart but low intensity in brain, and 3 HP = highly positive on MC-qPCR on heart and brain

$: This row contains dilutions of specimen (HF or FE) used.

FE -Filter eluate.

HF - Heart fluid.

UD-Undiluted.

N- Negative.

MAT- Modified agglutination test.

ELISA- Enzyme linked immuno sorbent assay.

IFAT- Indirect fluorescent antibody test.

### 3.4 Comparison of three serological methods relative to MC-qPCR

Because HF had a higher observed S/P% (in ELISA), titer (in MAT) and fluorescence (in IFAT) than FE, and a false negative result (ID-33) was obtained from FE (in MAT), we compared results using MAT (dilutions from 1:25 to 1:100), ELISA (dilution of 1:2) and IFAT (dilutions of 1:2 and 1:4) on HF. The results are presented in Table 2. All the test parameters (relative sensitivity, relative specificity, positive predictive value, negative predictive value and accuracy) were higher for ELISA and IFAT than for MAT (Table 3). Antibodies to *T. gondii* were detected in 16 (23.5%) of 68 wolverines using MAT, and 15 (22%) of 68 using ELISA or IFAT, respectively. In comparison to MAT (which incorrectly identified 4 tissue negative animals as positives), both ELISA and IFAT correctly identified more animals (15 vs. 12) that were positive in MC-qPCR (Table 2); thus ELISA and IFAT had higher relative sensitivity (94%) and relative specificity (100%) than MAT (75% and 92%). ELISA and IFAT showed excellent agreement (Kappa = 0.96) with MC-qPCR, whereas MAT showed only moderate agreement (Kappa = 0.67). As we did not test HF with dilutions lower than 1:25 using MAT, false negatives could occur due to low detection level of antibodies at higher dilution. Moreover, the highest dilution used was 1:100, and high concentration of antibodies (prozone phenomenon) can also lead to false negatives in MAT. Therefore, to rule out these two possibilities, we retested the HF samples, which on MAT were doubtful and had discrepant results with MC-qPCR, and dilutions of HF used were from 1:12.5 to 1:800. We obtained similar results.

**Table 2:** Detection of *Toxoplasma gondii* antibodies in wolverine using different serological methods (MAT, ELISA and IFAT). The infection status has been determined using the magnetic capture qPCR method.

	Number of tested samples		
	MC-qPCR		
Serological assay	Positive	Negative	Total
MAT			
Positive	12	4	16
Negative	4	48	52
Total	16	52	68
McNemar Test (p Value)	0.125	0.125	
IFAT			
Positive	15	0	15
Negative	1^a^	52	53
Total	16	52	68
McNemar Test (p Value)	1	–	
ELISA			
Positive	15	0	15
Negative	1^a^	52	53
Total	16	52	68
McNemar Test (p Value)	1	–	

MC-qPCR- Magnetic Capture-qPCR.

MAT- Modified agglutination test.

ELISA- Enzyme linked immuno sorbent assay.

IFAT- Indirect fluorescent antibody test.

a This wolverine had Cq value >35 on heart tissue, and was negative on brain tissue.

**Table 3:** Comparison of MAT, ELISA and IFAT to detect *Toxoplasma gondii* antibodies in naturally infected wolverines.^a^

Assay	K	p^mt^	Relative sensitivity	Relative specificity	PPV	NPV	Accuracy
MAT	0.67	1	75 (51–90)	92 (82–97)	75 (51–90)	92 (82–97)	88 (79–94)
IFAT	0.96	1	94(72–99)	100 (93.1–100)	100 (93.1–100)	98 (90–100)	99 (92–100)
ELISA	0.96	1	94 (72–99)	100 (93.1–100)	100 (93.1–100)	98 (90–100)	99 (92–100)

K- Kappa.

p^mt^- p value for McNemar test.

PPV- Positive Predictive Value.

NPV- Negative Predictive Value.

MAT- Modified agglutination test.

ELISA- Enzyme linked immuno sorbent assay.

IFAT- Indirect fluorescent antibody test.

a Parentheses have 95% CI.

## 4 Discussion

In the present study, DNA of *Toxoplasma gondii* was detected in heart of wolverines more commonly than in brain. This finding is similar to that reported on the bioassay of experimentally infected dogs and foxes (Dubey, 1985) (Dubey, 1983). Our study also found that heart had higher infection intensity (number of tachyzoite equivalents, TEG) than brain in naturally infected wolverines. This is an advantage of the MC-qPCR over bioassays which could not quantitatively evaluate the parasite burden in these tissues. In addition to higher positivity and parasite burden in hearts than brains, collecting heart (vs brain) is easier, less time consuming, requires less expertise, can provide tissue fluid for serology, and poses less risk of rabies. Therefore, heart can act as a better sampling site than brain for surveys to detect *T. gondii* in naturally infected wolverines. Our study and others demonstrate the utility of MC-qPCR to measure infection intensity for *T. gondii* as it provides both qualitative and quantitative results (Gisbert Algaba et al., 2018; Jurankova et al., 2014; Jurankova et al., 2015; Jurankova et al., 2013). Animal host species, infective stage, inoculation dose, time after inoculation (acute vs chronic phase) and genotype can affect the infection intensity and predilection sites (Gisbert Algaba et al., 2018; Jurankova et al., 2015; Koethe et al., 2015). Further work should include MC-qPCR on other tissues (such as skeletal muscle) to determine the predilection sites for *T. gondii* in wolverines, as well as genetic characterization of *T. gondii* to determine if different genotypes have different tissue predilections.

Results of our pilot study showed that both HF and FE could be used to detect anti-*T. gondii* antibodies, but FE could give false negatives. Indeed, one MC-qPCR positive wolverine (ID-33) was sero-positive with HF, but not with FE. Likewise, discrepant results between FE and serum using MAT were reported in cats (Bolais et al., 2017). Better performance of HF than FE (consistently higher S/P%, fluorescence intensity, and MAT titer) in the current study could be due to higher levels of anti- *T. gondii* antibodies in HF than FE. This may in part reflect that MAT was performed on relatively more diluted HF and FE, as we made two fold dilutions starting at 1:25 for MAT, but started at 1:2 dilutions for IFAT and ELISA. The ELISA kit used in this study suggests 1:10 dilution for serum and 1:2 for HF, and previous studies showed that serum was considered to have a higher level of anti-*T. gondii* antibodies than meat juice; therefore meat juice should be diluted less (1:2 vs. 1:10) than serum (Forbes et al., 2012; Wallander et al., 2015). Instruction manuals of commercial tests used herein indicate their intended use for serum, blood and meat juice, but have no information on their use for FE. Further, for a better evaluation of the suitability of HF and FE as matrices for serological tests, serum samples would have been needed as standard of comparison.

The manufacturers' recommended cut-off values and dilutions of HF [for MAT (cut-off: 1:25) and for ELISA (cut-off: S/P% greater than or equal to 50, dilution: 1:2)] seem appropriate for detecting anti-*T. gondii* antibodies in wolverines. Cut-off and dilutions of FE similar to HF can be used for ELISA and IFAT, but a choice for cut-off of FE in MAT requires further investigation. As our findings were based on small sample size (9 wolverines), further studies with large sample size are required.

Using MC-qPCR to determine infection status, ELISA and IFAT performed better than MAT in terms of relative sensitivity, relative specificity, PPV, NPV, and accuracy (Table 3). In comparison to MAT, ELISA and IFAT showed no false-positives (4 vs. 0) and fewer false-negatives (3 vs. 1), and therefore ELISA and IFAT had higher relative sensitivity and relative specificity. There were minimal chances that dilutions we used (1:25 to 1:100) were accountable for false negatives, but there is still a possibility that HF can be positive at lower dilution than the lowest dilution used (1: 12.5) in MAT; hence, further investigation is required. Previously, 1:10 dilution of meat juice has been suggested as a cut off using MAT to detect anti-*T. gondii* antibodies in experimentally infected pigs (Forbes et al., 2012), similar to the 1:12.5 dilution used in the current study.

There could be several reasons for false positives reported in MAT in our study. First, the *H. hammondi* positive control serum showed cross-reactivity with *T. gondii* in MAT in this and previous studies (Munday and Dubey, 1986; Weiland et al., 1979). This cross-reactivity could be due to some common antigens shared by two parasites (Araujo et al., 1984), and the positives we detected in MAT could have been infected with *H. hammondi*. Second, MC-qPCR could be giving a false negative, if the amount of parasite was below the detection limit of the test, or the parasite was present in tissues other than heart or brain. Previously, bioassays, DNA detection methods and serological tests or their combination have been used to evaluate performances of serological tests (Glor et al., 2013). Recently, serological tests have been compared in relation to MC-qPCR (Schares et al., 2018). MC-qPCR is more sensitive than conventional PCR techniques, but may be less sensitive than a cat bioassay, which is the gold standard test for detection of *T. gondii* (Dubey, 2010). This comparison has not been done directly on the same samples. Moreover, higher sensitivity and specificity of the MC-qPCR for detection of *T. gondii* (89.2% and 100%) was reported in experimentally infected pigs(Opsteegh et al., 2010). In a recent study, comparison between mouse bioassay and MC-qPCR for detection of *T. gondii* in experimentally infected pigs showed that the MC-qPCR was more sensitive (sensitivity = 94%) than mouse bioassay (86%), whereas both methods had equal specificities (100%) (Gisbert Algaba et al., 2017). Therefore, MC-qPCR provides comparable results with that of mouse bioassay. Since MC-qPCR has a low limit of detection of 4.45 tachyzoites/ g tissue (Bachand et al., 2018), we used both heart and brain (most common predilection sites in other species), and ELISA and IFAT showed no positives on those four samples, it is more likely that MAT is giving a false positive.

In comparison to MAT, ELISA and IFAT are less laborious and time consuming and results are available on the same day. However, they require special instruments such as an ELISA reader and fluorescent microscope. Another disadvantage can be the prerequisite of species-specific conjugate, which are not available commercially mostly for wildlife. This is likely why the MAT (or DAT), which does not require species-specific conjugate, has been the most widely used test to detect anti-*T. gondii* antibodies in wildlife. However, this is addressed in the current study by using a protein A/G conjugate in both ELISA and IFAT, which binds to the IgG of various mammalian species, and has successfully been used for detection of anti-*T. gondii* antibodies in several species (Addison and Boles, 1978; Al-Adhami and Gajadhar, 2014; Schaefer et al., 2012). This is the first study to use protein A/G in IFAT to detect anti-*T. gondii* antibodies in wolverines. Our results add to the evidence that mammalian generalized protein A/G conjugate can be used in serological tests for multiple species for which commercial conjugates are not available. In summary, ELISA and IFAT were in excellent agreement, and either can be used for future surveys; in addition, ELISA results are automatically read, making them more objective than MAT or IFAT results.

## 5 Conclusion

Heart was a better sampling site than brain for detection of *T. gondii* DNA in wolverines due to the following findings: (1) a higher parasite burden detected, (2) more practical sampling site than brain, and (3) concurrent availability of heart fluid that can be used for comparative serological testing. However, further studies are required to identify other predilection or sampling sites. As serum from wild animals is usually unavailable, heart fluid can be a good candidate matrix to detect anti-*T. gondii* antibodies in wolverines using ELISA, IFAT and MAT, but cut-off values and false positives (low specificity) need to be further investigated for MAT. Based on our findings, HF was preferred over FE to avoid false negatives. Further comparison between HF and FE should be studied with larger sample size. We suggest evaluating the performance of commercially available kits before their usage to detect *T. gondii* antibodies in wildlife species. As experimentally infected animals are generally lacking for wildlife species, sensitive tests to determine infection status of naturally infected animals should be used; for example, MC-qPCR, when bioassay is not feasible (frozen tissues, large scale studies) or desirable (animal research ethics).
